# Fetal *RHD* Genotyping Using Real-Time Polymerase Chain Reaction Analysis of Cell-Free Fetal DNA in Pregnancy of RhD Negative Women in South of Iran 

**DOI:** 10.22074/ijfs.2016.4770

**Published:** 2016-04-05

**Authors:** Leili Moezzi, Zeinab Keshavarz, Reza Ranjbaran, Farzaneh Aboualizadeh, Abbas Behzad-Behbahani, Masooma Abdullahi, Amin Ramezani, Alamtaj Samsami, Sedigheh Sharifzadeh

**Affiliations:** 1 Diagnostic Laboratory Sciences and Technology Research Center, School of Paramedical Sciences, Shiraz University of Medical Sciences, Shiraz, Iran; 2 Student Research Committee, Shiraz University of Medical Sciences, Shiraz, Iran; 3 School of Advanced Medical Science and Technology, Shiraz University of Medical Sciences, Shiraz, Iran; 4 Department of Obstetrics and Gynecology, Medical School, Shiraz University of Medical Sciences, Shiraz, Iran

**Keywords:** Prenatal Diagnosis, Real-Time Polymerase Chain Reaction, Cell-Free Fetal DNA

## Abstract

**Background:**

Maternal-fetal RhD antigen incompatibility causes approximately 50% of clinically significant alloimmunization cases. The routine use of prophylactic anti-D immunoglobulin has dramatically reduced hemolytic disease of the fetus and newborn. Recently, fetal *RHD* genotyping in RhD negative pregnant women has been suggested for appropriate use of anti-D immunoglobulin antenatal prophylaxis and decrease unnecessary prenatal interventions.

**Materials and Methods:**

In this prospective cohort study, in order to develop a reliable and non-invasive method for fetal *RHD* genotyping, cell free fetal DNA (cffD- NA) was extracted from maternal plasma. Real-time quantitative polymerase chain reaction (qPCR) for detection of *RHD* exons 7, 5, 10 and intron 4 was performed and the results were compared to the serological results of cord blood cells as the gold standard method. *SRY* gene and hypermethylated Ras-association domain family member 1 (*RASSF1A*) gene were used to confirm the presence of fetal DNA in male and female fetuses, respectively.

**Results:**

Out of 48 fetuses between 8 and 32 weeks (wks) of gestational age (GA), we correctly diagnosed 45 cases (93.75%) of *RHD* positive fetuses and 2 cases (4.16%) of the *RHD* negative one. Exon 7 was amplified in one sample, while three other *RHD* gene sequences were not detected; the sample was classified as inconclusive, and the RhD serology result after birth showed that the fetus was RhD-negative.

**Conclusion:**

Our results showed high accuracy of the qPCR method using cffDNA for fetal *RHD* genotyping and implicate on the efficiency of this technique to predict the competence of anti-D immunoglobulin administration.

## Introduction

Rh is systematically the most polymorphic blood group and clinically the most important group after ABO. RH complex is formed by two highly homologous *RHD* and RHCE genes, both of which are located on chromosome 1, and consist of 10 exons (1). Although complete deletion of *RHD* gene is found to be dominant in Caucasian D-negatives, there is a large diversity in other populations especially in Japanese and African blacks (2). About 66% of the RhD-negative African population carry a non-functional *RHD* gene named *RHD* pseudogene (*RHD* ψ), and 15% of RhD-negative Africans have a special rearrangement of *RHD* and *RHCE* genes, named the hybrid allele *RHD-CE-Ds* (3, 4).

The D-negative phenotype has a wide range frequency in different ethnic populations. With regards to literatures, the frequency of RhD-negative is 3-7% in Africans,15-20% in Caucasians and less than 1% (0.3-0.5) in the far east (5). In a study conducted in Fars province of Iran, the frequencies of RhD negative phenotype were 13.05 and 9.62% in 1982 and 2001, respectively (6). Maternal- fetal RhD antigen incompatibility causes approximately 50% of clinically significant maternal alloimmunization cases. Since1960s, the routine use of prophylactic anti-D immunoglobulin has dramatically decreased the hemolytic disease of the fetus and newborn (7). Fetal *RHD* genotyping in RhD negative antenatal women can be effective for the appropriate use of anti-D antenatal prophylaxis, facilitating to reduce unnecessary prenatal interventions. In an immunized pregnant woman, the prediction of fetal RhD blood group is helpful for the appropriate management of the pregnancy and avoiding unnecessary invasive tests. At the same time, this reduces the concerns about the pregnancy outcome (8-11). For many years, prenatal diagnosis has been performed by chorionic villus sampling (CVS) and amniocentesis. These invasive tests increase the risk of feto-maternal hemorrhage and enhance the severity of alloimunization. In addition, performing these tests before 11 weeks (wks) of pregnancy is not recommended. Although CVS provides the result in the first trimester, it is associated with higher risk of miscarriage than amniocentesis: 1in 100-200 vs. 1 in 200-400, respectively (12-14).

Detection of low fetal DNA concentration in maternal plasma (3% in early to 6% in late pregnancy) and distinguishing cffDNA from maternal DNA are the two major challenges that limit the use of cffDNA for non-invasive prenatal tests (NIPT) (18, 19).

Different methods have been used to confirm the presence of fetal DNA in maternal plasma, in previous studies. The most common system is to trace *SRY* sequence in maternal plasma; it also provides the possibility of determining the sex of the fetus, but this strategy is not applicable for female fetuses (4). Another possible method is an evaluation of polymorphic microsatellites and insertion/deletion markers in maternal plasma and buffy coat. Failure to detect a specific allele in maternal buffy coat together with its presence in maternal plasma is the basis for diagnosis. Such methods are not able to provide sufficient information and also have low sensitivity (20, 21). In a recent method, introduced as a universal marker, tracking is performed based on different methylation of the *RASSF1A* gene in maternal and fetal DNA (8, 22). The aim of our study was to set up a novel reliable protocol for non-invasive determination of fetal RhD status using cffDNA extracted from maternal plasma.

## Materials and Methods

In this prospective cohort study, the plasma samples were collected from 50 RhD-negative women with singleton pregnancy at Hafez Hospital, Shiraz, Iran. Gestational age was between 8 and 32 wks, based on the last menstrual period (LMP). 10 blood samples were taken at 8-16 wks of gestation age (GA), 35 samples at 17-28 wks and 5 samples at ≥28 wks of GA. The participants were healthy women without any serious pregnancy complications, and their husbands were serologically RhDpositive.

### Sample preparation

Peripheral blood samples were collected in a 6 ml tube containing Ethylenediaminetetraacetic acid (EDTA, INTERLAB Laboratory Products, Turkey) and processed within 6 hours. The samples were centrifuged at 2000 ×g for 10 minutes to separate the plasma, which were subsequently centrifuged at 3000 ×g for 10 minutes. The supernatants were then separated and stored at -80°C for further processing.

### DNA extraction

QIAamp DNA Blood Mini Kit (Qiagen, Hilden, Germany) was used to extract cffDNA from plasma with minor modification. DNA was isolated from 200 μl of plasma according to manufacturer’s instruction, but eluted in a final volume of 30 μl Buffer AE (INTERLAB Laboratory Products, Turkey). To minimize the risk of contamination, DNA was isolated under laminar airflow and aerosol-resistant tips were used.

### Real-time polymerase chain reaction

Real-time PCR was performed on Rotor-Gene Q (Qiagen, USA) using SYBR Green Master Mix (2x Maxima SYBR Green/ROX qPCR Master Mix, Thermo Scientific, Lithuania). To determine the fetal RhD status, the presence of *RHD* exons 5, 7, 10 and intron 4 were evaluated. The AlleleID 7.5 primer software (PREMIER Biosoft, USA) was employed to design *SRY* primers using the *SRY* gene sequence obtained from GenBank nucleotide database (accession number: L08063). All other primers were selected according to previous studies presented in the Table 1 (23-28).

All quantitative polymerase chain reaction (qPCR) reactions were performed in a final volume of 25 μl containing 5 μl of DNA. The final concentration of primers in each qPCR reaction was 300 nmol.L-1. The qPCR cycling condition was two-step holding temperatures: 50°C for 2 minutes, 95°C for 10 minutes followed by 50 cycles of 94°C for 60 seconds, 55°C for 60 seconds, and 72°C for 60 seconds. Two replicates were performed for the tested gene.

The fetuses were labeled either as a D-positive, when all *RHD* target sequences (exons 5, 10, 7 and intron 4) were properly amplified, or D-negative, when no amplification signal was detected. Fetuses were predicted to be inconclusive when one, two or three specific *RHD* sequences were amplified. The cycle threshold (Ct) values of 30-42 were considered positive.

### Quality control

10-fold serial dilutions were prepared to determine the sensitivity of the test, the quality of primers, and qPCR reagents using DNA extracted from plasma of a male human. To rule out the possible contamination, positive controls, negative controls and no-template controls (NTCs) were also included in each PCR run, using sterile H2O. The *β-globin* gene, as a reference gene, was tested to confirm the presence of cell free DNA (cfDNA).

**Table 1:** Sequences of PCR primers for real time PCR assays


Target genes	Sequence 5' to 3'

*RHD (intron 4)*	F: GATGACCAAGTTTTCTGGAAA
	R: CATAAACAGCAAGTCAACATATATACT
*RHD (exon 5)*	F: CGCCCTCTTCTTGTGGATG
	R: GAACACGGCATTCTTCCTTTC
*RHD (exon 7)*	F: CTCCATCATGGGCTACAA
	R: CCGGCTCCGACGGTATC
*RHD (exon 10)*	F: CCTCTCACTGTTGCCTGCATT
	R: AGTGCCTGCGCGAACATT
*SRY*	F: AATTGGCGATTAAGTCAA
	R: TGTATTCATTCTCAAGCAA
*RASSF1A*	F: AGCCTGAGCTCATTGAGCT
	R: ACCAGCTGCCGTGTG
*β-globin*	F: GTGCACCTGACTCCTGAGGAGA
	R: CCTTGATACCAACCTGCCCAG


PCR; Polymerase chain reaction.

### Validating presence of the cell-free fetal DNA in RhD-negative female fetuses

*SRY* gene was used for all samples to confirm the presence of cffDNA. In the predicated samples as RhD negative female, the presence of hypermethylated *RASSF1A* gene was also tested. Investigations show that the RASSF1 gene promoter is hypermethylated in DNA with placenta origin, but hypomethylated in maternal DNA (29). The cfDNA samples were initially treated with BstUI, a methylation-sensitive restriction enzyme. At this experiment, digestion reactions contained 0.5 μg DNA and 5 U BstUI restriction enzyme (New England Biolabs, England) were incubated at 60ºC for 2 hours followed subsequently by adding to qPCR reactions. Each run included three different controls: undigested non-pregnant control (DNA from a non-pregnant woman), digested non-pregnant control (DNA from a non-pregnant woman), and undigested pregnant control (DNA obtained from a pregnant woman).

### RhD phenotype of newborns

Blood samples were collected at birth from cord blood. The direct agglutination test was carried out with anti-RhD reagents (CinnaGene, Iran). The concordance of test was determined by comparing the data from the prenatal genetic tests with serological results obtained from cord blood.

### Statistical analysis

The this study, simple random sampling (SRS) method was used to collect clinical samples. As analytical values, limit of detection in qPCR test in clinical samples was defined. Using serology and neonate sex, as two gold standard test to respectively confirm *RHD* and *SRY* gene results, the diagnostic sensitivity, specificity and concordance were reported. Roc curve analysis was employed and P value>0.05 was reported as statistically significant level. All the statistical analyses were performed by SPSS, version 16.0.(Ltd, Hong Kong)

### Ethical considerations

All procedures for this study were approved by the Ethics Committee (ec-p-90-3311) of Shiraz University of Medical Sciences (Shiraz, Iran). Informed consent was obtained from pregnant women who participated in this research project.

## Results

Non-invasive prenatal determination of fetal RhD status, as well as gender analysis, was performed in 48 cases of RhD-negative pregnant women, while their husbands were RhD-positive. The mean gestational age was 26 wks at the time of blood sampling (ranging from 8 to 32 wks). Serological tests were performed on the cord blood sample, and the fetal gender was confirmed after delivery.

The minimum detection level of DNA in clinical samples was 4.2 (pg/μl). qPCR was performed on the samples in duplicates and the results were interpreted as positive, provided detection of the specific amplicons in both replicates.

**Fig 1:**
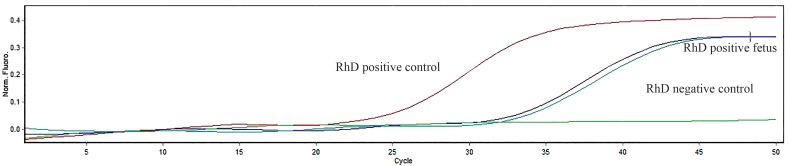
Real-time quantitative polymerase chain reaction (PCR). Amplification plots using real-time quantitative PCR for the *RHD* (exon 7) gene. Positive control; DNA from a RhD positive woman, Samples; Result observed from *RHD* negative women holding RhD positive fetuses, Negative control; Result observed from RhD negative women.

**Fig 2:**
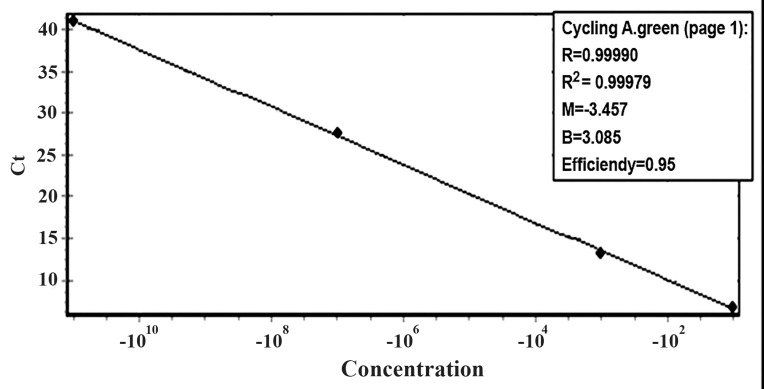
Real time standard curve of *RHD* exon 7 gene using 10-fold serially diluted samples. The plot shows the relationship between Ct value and DNA concentration. R; Correlation coefficient, R^2^; Coefficient of determination, M; M-estimation, B; Beta coefficient and Ct; Cycle threshold.

Analysis of the standard curves of qPCR demonstrated a wide dynamic range and high efficiency for the investigated genes (Fig .1). The Ct value ranges in maternal plasma of clinical samples are presented in the Table 2. Figure 2 represents the qPCR results of *RHD* exon 7 in the controls and clinical samples.

Analysis of two fetuses were not terminated and they were excluded from our samples due to abortion and hydrops fetalis. Following the amplification of all *RHD* gene target sequences, the fetuses were classified in different RhD positive groups. Out of 48 samples, the results of 45 cases (93.75%) were determined as RhDpositive, and 2 cases (4.16%) were detected as RhD-negative. Exon 7 was amplified in one sample (2.08%) while no signal was determined for the other three *RHD* gene fragments. The results obtained from this sample were considered to be inconclusive (Table 3) while serologic finding distinguished the fetus as Rh negative.

Serology of the cord blood indicated 45 RhD positive neonates (93.75%) and 3 RhD negative ones (6.25%). Based on a prenatal test for *SRY* gene, 5 cases (10.41%) were predicted to be male and 43 cases (89.58%) female (Table 3). There was complete concordance between *SRY* qPCR results and neonate gender after delivery. The Diagnostic concordance of the test was 100% for the *SRY* gene and 97.91% for the *RHD* gene (Table 4).

Three samples out of the 48 showed negative qPCR result for *RHD* and *SRY* genes. In order to confirm the presence of fetal DNA, *RASSF1A* qPCR was performed after methylation-sensitive restriction enzyme digestion. The obtained result confirmed the presence of cffDNA in all three samples.

**Table 2:** qPCR efficiencies, linear correlations (R2) of standard dilutions, and ranges of Ct value for the tested genes


Target genes	qPCR efficiency (%)	R^2^	Ct value ranges in clinical samples

*β-globin*	0.95	0.99	30-36.2
*RHD* intron 4	0.91	0.99	33.64-41.83
*RHD* exon 5	0.91	0.99	33.20-41.77
*RHD* exon 7	0.95	0.99	35.99-41.32
*RHD* exon 10	0.91	0.99	32.78-41.49
*SRY*	0.92	0.99	35.83-41.60


**Table 3:** Fetal RHD and SRY genotyping results by qPCR and neonatal RhD phenotype and sex


Sample no.	Maternal RhD phenotype	Fetal genotyping in maternal plasma					Neonate RhD phenotype	Neonate sex
		*RHD* exon 5	*RHD* exon 7	*RHD* exon 10	*RHD* intron 4	*SRY*		

1	Neg	Pos	Pos	Pos	Pos	Neg	Pos	♀
2	Neg	Pos	Pos	Pos	Pos	Neg	Pos	♀
3	Neg	Pos	Pos	Pos	Pos	Neg	Pos	♀
4	Neg	Pos	Pos	Pos	Pos	Neg	Pos	♀
5	Neg	Pos	Pos	Pos	Pos	Neg	Pos	♀
6	Neg	Pos	Pos	Pos	Pos	Neg	Pos	♀
7	Neg	Pos	Pos	Pos	Pos	Neg	Pos	♀
8	Neg	Pos	Pos	Pos	Pos	Neg	Pos	♀
9	Neg	Pos	Pos	Pos	Pos	Neg	Pos	♂
10	Neg	Pos	Pos	Pos	Pos	Neg	Pos	♀
11	Neg	Neg	Pos	Neg	Neg	Neg	Neg	♀
12	Neg	Pos	Pos	Pos	Pos	Pos	Pos	♂
13	Neg	Pos	Pos	Pos	Pos	Neg	Pos	♀
14	Neg	Pos	Pos	Pos	Pos	Neg	Pos	♀
15	Neg	Pos	Pos	Pos	Pos	Pos	Pos	♂
16	Neg	Pos	Pos	Pos	Pos	Neg	Pos	♀
17	Neg	Pos	Pos	Pos	Pos	Neg	Pos	♀
18	Neg	Pos	Pos	Pos	Pos	Neg	Pos	♀
19	Neg	Neg	Neg	Neg	Neg	Neg	Neg	♀
20	Neg	Pos	Pos	Pos	Pos	Neg	Pos	♀
21	Neg	Pos	Pos	Pos	Pos	Neg	Pos	♀
22	Neg	Pos	Pos	Pos	Pos	Neg	Pos	♀
23	Neg	Pos	Pos	Pos	Pos	Neg	Pos	♀
24	Neg	Pos	Pos	Pos	Pos	Neg	Pos	♀
25	Neg	Pos	Pos	Pos	Pos	Neg	Pos	♀
26	Neg	Pos	Pos	Pos	Pos	Neg	Pos	♀
27	Neg	Pos	Pos	Pos	Pos	Neg	Pos	♀
28	Neg	Pos	Pos	Pos	Pos	Neg	Pos	♀
29	Neg	Pos	Pos	Pos	Pos	Neg	Pos	♀
30	Neg	Pos	Pos	Pos	Pos	Neg	Pos	♀
31	Neg	Pos	Pos	Pos	Pos	Neg	Pos	♀
32	Neg	Pos	Pos	Pos	Pos	Neg	Pos	♀
33	Neg	Pos	Pos	Pos	Pos	Neg	Pos	♀
34	Neg	Neg	Neg	Neg	Neg	Neg	Neg	♀
35	Neg	Pos	Pos	Pos	Pos	Neg	Pos	♀
36	Neg	Pos	Pos	Pos	Pos	Neg	Pos	♂
37	Neg	Pos	Pos	Pos	Pos	Neg	Pos	♀
38	Neg	Pos	Pos	Pos	Pos	Neg	Pos	♀
39	Neg	Pos	Pos	Pos	Pos	Neg	Pos	♀
40	Neg	Pos	Pos	Pos	Pos	Neg	Pos	♀
41	Neg	Pos	Pos	Pos	Pos	Neg	Pos	♀
43	Neg	Pos	Pos	Pos	Pos	Neg	Pos	♀
44	Neg	Pos	Pos	Pos	Pos	Neg	Pos	♀
45	Neg	Pos	Pos	Pos	Pos	Neg	Pos	♀
46	Neg	Pos	Pos	Pos	Pos	Pos	Pos	♂
47	Neg	Pos	Pos	Pos	Pos	Neg	Pos	♀
48	Neg	Pos	Pos	Pos	Pos	Neg	Pos	♀


qPCR; Qantitative polymerase chain reaction.

**Table 4:** Diagnostic measures between genotyping and phenotyping


	RHD gene	SRY gene

Concordance	97.91% (47/48)	100%
Sensitivity	100%	100%
False-negatives	-	-
Specificity	100%	100%
False-positives	-	-


## Discussion

Our findings confirmed the reliability of non-invasive prenatal testing to predict the fetal RhD status. This prediction can be helpful to determine the necessity of close fetal monitoring and the need of more invasive procedures in isoimmunized mothers. Another positive outcome of fetal *RHD* prediction is preventing unnecessary anti-D immunoglobulin injection in nonisoimmunized mothers with RhD negative fetuses. A study, performed in UK, showed that 38% of RhD-negative pregnant women bear RhD-negative fetus. Therefore, employing non-invasive prenatal test can reduce the cost of the health care system and risks of viral infection pertaining to anti-D administration (30).

Based on previous experiences, there are several important steps in developing NIPT including: blood sample preparation (31), cffDNA extraction (32) and confirming presence of cffDNA (33). Additionally, regarding the reported genetic diversity at RH system within different ethnic groups, selection of *RHD* gene sequences for qPCR test and defining specific rules for interpretation of genotype are inevitable (34). Therefore, we developed a novel non-invasive prenatal diagnostic test using cffDNA in our laboratory, to evaluate the fetal RhD status within pregnant populations obtained from south of Iran. Previous studies have recommended the use of at least 2 *RHD* specific regions to avoid false positive results, although using multi-sequences to trace *RHD* diversity have recently become more widespread. In this study, all samples were tested for the presence of *RHD* exon 10 and intron 4 to distinguish between two homologous *RHD* and RHCE genes. In addition, exon 5 analysis was applied to identify the point mutations leading to *RHD*ψ. Moreover, in order to cover different types of partial D categories, especially DVI partial D as the most common hybrid *RHD-CE-Ds*, selected areas of *RHD* gene (intron 4, exons 5, 7 and 10) were included (35-38).

In this study, the false negative and false positive results were not observed, except in one sample that *RHD* exon 7 was amplified, while intron 4, exon 5 and exon 10 did not identify. This case was classified in the inconclusive group, and serology results showed the fetus as RhD negative. The possible cause of these findings was an *RHD* variant gene in the mother or fetus, but there was no access to maternal or newborn DNA for subsequent analysis. Comparison of three previously published studies (39, 35, 13) showed similar findings to our results.

Although the presence of fetal DNA was not confirmed in most of previously published studies (40, 41), our strategy was using *SRY* gene for all the samples and in cases that were negative for *SRY* and *RHD* genes, hypermethylation of RASSF1 gene by BstUI restriction enzyme was evaluated. In order to avoid false-negative results followed by mismanagement of the pregnancy, analyzing *RASSF1A* gene is essential for the cases with *RHD* negative female fetuses.

## Conclusion

In this study, diagnostic concordance of the predicted fetal gender (100%) and RhD status (97.91%) from free fetal DNA in the maternal plasma of 48 *RHD* negative women were obtained. With regards to observing no different Rh variants in this experiment, a large study from different region of our countryIranis suggested. Thus, this study can be helpful to find possible *RHD* variants as well as the cause of inconclusive cases. Conducting larger-scale studies will be the first step in establishing a guideline for running non-invasive *RHD* genotype testing on all *RHD* negative mothers in Iran.
